# “Exploring the intersection of albinism and Trichilemmal carcinoma: A case of bilateral trichilemmal carcinoma in the axilla”

**DOI:** 10.1016/j.ijscr.2025.110926

**Published:** 2025-01-23

**Authors:** Musab A. Dfallah, Ahmed M. Elamin, Mohammed A. Gerib, Khalid E. Ahmed, Abdalla A. Arabi, Osama Abdelmutaal Idris Mutwali

**Affiliations:** a Omdurman Islamic university, Sudan; b MD plastic surgery SMSB, Sudan; c U of K, Sudan; d Gezira university, Sudan; e National Ribat University, Sudan; f Red sea university, Sudan

## Abstract

**Introduction and background:**

Albinism is an autosomal recessive disorder characterized by the absence of melanin in the skin, albinism has a prevalence of 1 in 1000 in sub-Saharan Africa, with a lack of melanin increased risk of skin malignancy. Trichlemmal carcinoma is a rare malignant adnexal tumor mostly in sun-exposed areas.

**Case presentation:**

We are presenting a case of a 35-year-old albino patient with a previous history of squamous cell carcinoma with full recovery. Presented with bilateral axillary masses with no other finding in history or examination. Short period deference between the two tumors with rapidly growing course. Both tumors were excised but in different operations, one covered with latissimus dorsi and the other primarily. Both tumor histopathologies revealed trichilemmal carcinoma with a free tumor margin.

**Discussion:**

Albinism, caused by tyrosine deficiency and characterized by a lack of melanin, significantly increases susceptibility to ultraviolet (UV) radiation and skin cancers, particularly squamous cell carcinoma (SCC) and basal cell carcinoma (BCC). Trichlmmal carcinoma TLC, an uncommon malignancy from hair follicle structures, is notably rare, comprising <0.005 % of adnexal carcinomas. Post-surgical analysis confirmed TLC with clear margins, emphasizing the need for vigilant follow-up to monitor for recurrence or new lesions. Strong sun protection measures—such as protective clothing and broad-spectrum sunscreen—are essential for individuals with albinism.

**Conclusion:**

This case illustrates the necessity for healthcare providers to be aware of the diverse skin cancer risks in patients with albinism and to implement tailored preventive strategies.

## Highlights

• Albinism is autosomal recessive disorder characterized by absence of melanin in skin, albinism has prevalence 1 in 1000 in sub-Saharan Africa, with lack melanin increased risk of skin malignancy. Trichlemmal carcinoma is rare malignant adnexal tumor mostly in sun exposed area.
• we are presenting case of 35 years old albino patient with previous history of squamous cell carcinoma with full recovery. Presenting with bilateral axillary masses with short period deference in between with rapidly growing course. Both tumor were excised but in different operation one covered with litassmus dorsi and the other primarily. Both tumor histopathology reviled trichlmmal carcinoma with free tumor margin.
• This case illustrates the necessity for healthcare providers to be aware of the diverse skin cancer risks in patients with albinism and to implement tailored preventive strategies. Future research should explore genetic predispositions and environmental influences on rare skin cancers in this vulnerable population. Overall, while albinism is primarily associated with pigmentation issues, its implications for skin cancer risk are significant and warrant proactive management.

## 1 Introduction

Albinism comprises a heterogeneous group of inherited autosomal recessive disorders characterized by the absence or dysfunction of the tyrosinase enzyme, which is essential for converting tyrosine to the melanin precursor dihydroxyphenylalanine (DOPA). This enzymatic deficiency results in diminished or absent melanin synthesis in melanocytes in the skin, hair, and eyes [1]. The global incidence of albinism is 1:20,000 individuals, with a higher prevalence in sub-Saharan Africa, ranging from 1:15,000 to 1:1000 [2]. The absence or dysfunction of melanin in individuals with albinism renders them particularly vulnerable to the harmful effects of ultraviolet radiation. This increased susceptibility predisposes them to a range of cutaneous pathologies, including skin cancers such as squamous cell carcinoma and basal cell carcinoma [3,4]. All individuals with albinism face a significant risk of developing squamous cell carcinoma in sun-exposed areas.in sub-Saharan Africa, dark colored with albinism are far 1000 times more likely to develop skin cancer [5].

Trichilemmal carcinoma (TLC) is a rare malignant adnexal neoplasm, accounting for <0.005 % of adnexal carcinomas, characterized by features indicative of outer root sheath differentiation, as well as atypical clear cell morphology [6] TLC usually develop in sun-exposed area and related to sun exposure. We present a case of bilateral axillary trichilemmal carcinoma with recurrence in a patient with albinism, highlighting the unique clinical challenges and implications associated with this rare presentation. The work has been reported in line with the SCARE criteria. [7]

## 2 Case presentation

**Fig. 1:**
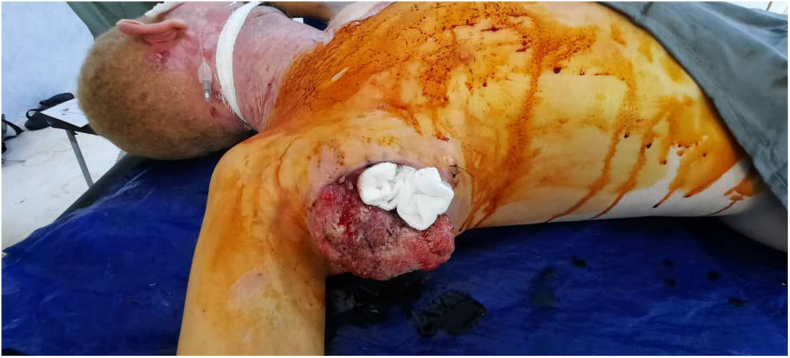

**Fig. 2:**
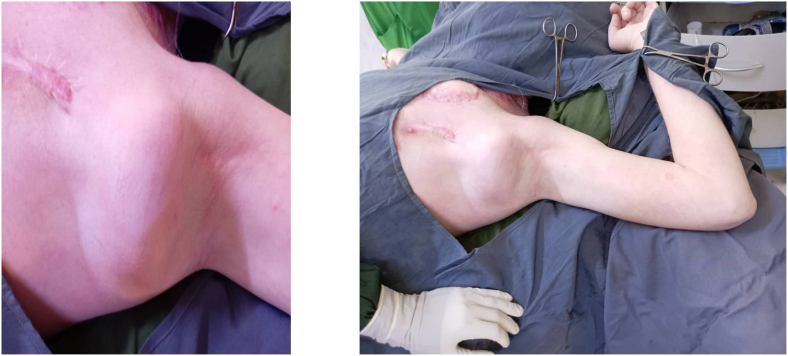

**Fig. 3:**
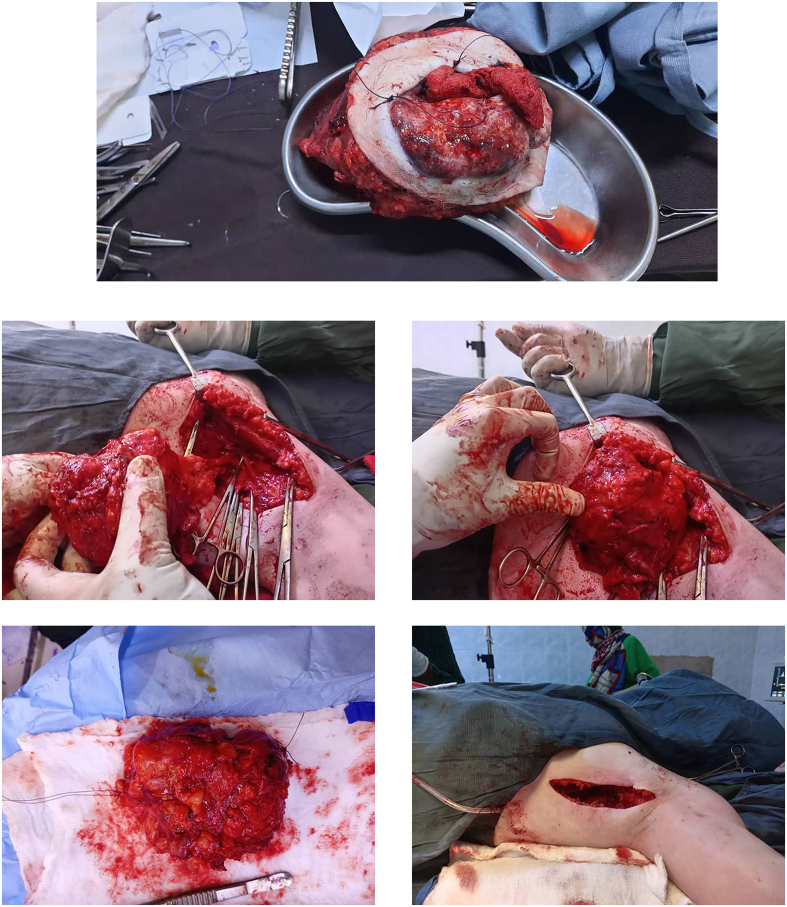

**Fig. 4:**
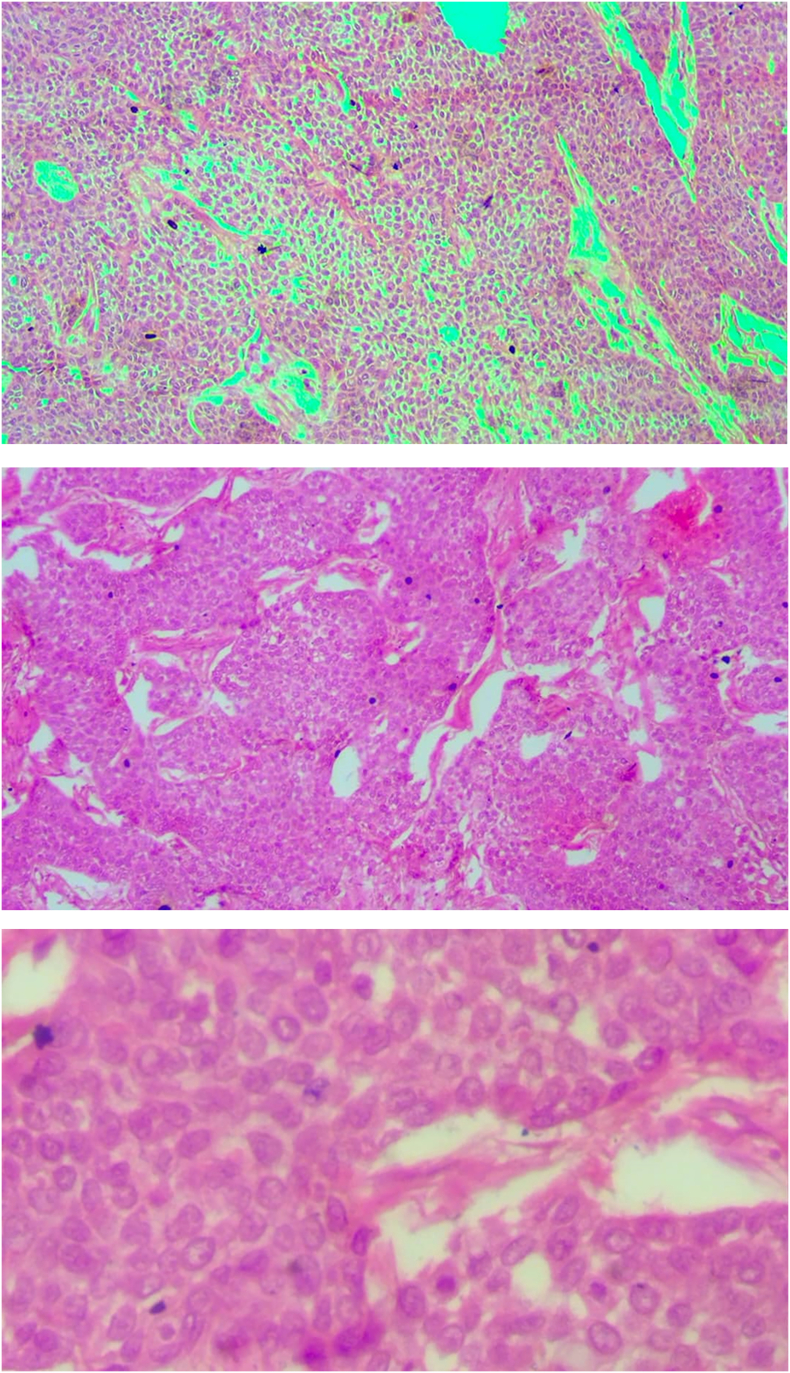

A 35-year-old Sudanese male working accountant with a known history of albinism and a family history of the condition, including an affected sibling and son, has no history of extensive sun exposure. With a previous history of squamous cell carcinoma in the chest, it was excised in early 2021 with complete recovery (Fig. 1, Fig. 2, Fig. 3, Fig. 4).

Presented in late December 2023 with a rapidly growing right axillary swelling that had developed over the past two months, increasing from the size of a lemon to that of an orange. The mass was not preceded by any trauma or radiation exposure, was non-tender, and restricted movement in the affected area.

On examination, a 15 × 20 cm mass was identified in the right axilla. The mass had an ulcer 10 × 8 cm on its surface, was firm in consistency, rounded in shape, and was not adherent to the overlying skin or underlying structures. During the examination, an additional mass was discovered in the left axilla, measuring 10 × 8 cm. This mass was firm, fixed to the skin, but not attached to underlying structures, and was also non-tender with no associated skin changes. There is no other swelling in the axilla; distal pulsation is intact—no movement or sensation deficits in the affected limb. Chest and abdominal examinations were normal, with another swelling in the axilla; distal pulsation is intact—no movement or sensation deficits in the affected limb. Chest and abdominal examinations were normal.

The patient underwent surgical intervention in early January for excision of the right axillary tumor. Preoperative investigations were within normal limits. The tumor was excised, and the resulting defect was covered using a latissimus dorsi myocutaneous flap. The patient fully recovered from anesthesia. A post-operative examination of the flap indicated normal findings. The patient was admitted to the ward for five days to receive intravenous antibiotics and to facilitate monitoring of the flap. Upon completion of the treatment, the patient was discharged with instructions for follow-up and dressing changes.

Following three weeks, the patient underwent a second surgical procedure for the removal of the left axillary tumor, which was closed primarily. The patient was discharged on day 1 post-operatively with no problems. By the time the patient underwent the second operation, the first wound had completely healed.

One month after the second surgery the wounds of the second operation were completely healed, and the patient returned for follow-up after both surgical sites had healed. Histopathological analysis of the excised tissues from both axillary masses revealed malignant adnexal skin tumors consistent with trichilemmal carcinoma. The microscopic finding revealed an infiltrating tumor with multiple connections to the epidermis and pushing the margins. The tumor is surrounded by fibrotic stroma and shows peripheral palisading along with retraction artifacts the tumor cells are rounded to polygonal with clear cytoplasm. Clear margins were noted on both specimens. The patient later on was advised to have good sun cover reduce sun exposure and use the sun screen to avoid any subsequent malignancy in the feature. The patient was followed up for 1 year with no complication or recurrence.

## 3 Discussion

We are presenting a rare case report of bilateral axillary trichilemmal carcinoma in a patient with albinism. The contradicting combination of Africa and albinism is that the first has a lower risk for skin cancer and the latter has increased risk. The usual suspects of skin cancer is squamous cell carcinoma, basal cell carcinoma malignant melanoma and the most common in African albino is squamous cell carcinoma [8,9]. Skin cancer in African non-albino is more common in the lower extremities followed by anorectal, while in albino Africans in the head neck, and upper extremities. The presence of trichelammal carcinoma was reported in albino in which were in the eyelid [10,11], given that sun exposure is a risk for the development of skin malignancy in albinism and also risk for developing trichelammal carcinoma yet not having a history of extensive sun exposure the cumulative role of solar radiation plays a major role in cancar in albinos [12]. Trichelammal carcinoma has no gender dominance with nearly equal distribution of cases between genders in concern to age the mean age of tumor presentation is between 60 and 80 years, yet in this case, the patient age is below this in which can be due to the predisposition of albino to malignancy [13]. The trichilammal carcinoma has no typical presentation this can be due to its co-occurrence with other diseases. So the clinical presentation of trichelammal carcinoma is not reliable for diagnosis [14]. Specifically in this case in which there is a risk for other skin malignancy and dominates presentation. The presentation of the tumor in bilateral has not been seen in previous cases in the literature and this may be due to a lack of studies that link the two presentations together in which albinism can present by higher risk for developing trichelammal carcinoma. Currently, the differential diagnosis of trichilemmal carcinoma (TLC) primarily depends on hematoxylin and eosin (HE) staining. While periodic acid-Schiff (PAS) staining is also frequently used, it should not be solely relied upon for diagnosing TLC, as it can yield positive results in clear-cell squamous cell carcinoma (SCC) and clear-cell basal cell carcinoma (BCC) due to its ability to highlight glycogen. In cases where the differential diagnosis is challenging, special stains can provide additional helpful clues [15,16]. It is important to recognize that the pathological criteria for diagnosing trichilemmal carcinoma (TLC) can differ among various authors. Additionally, due to the similarities in cellular origin and pathological characteristics between TLC and other skin tumors, such as squamous cell carcinoma (SCC), some dermatologists and pathologists argue that TLC may represent a clear-cell variant of SCC rather than a distinct disease entity (17). The risk of recurrence of trichielemmal carcinoma is <10 % with complete excision of the margin in the absence of lymph node involvement.

In Conclusion, while the case of bilateral axillary trichilemmal carcinoma in a patient with albinism is rare, it serves as a reminder of the complexities involved in managing skin cancers in this population. Continuous education on sun safety, regular dermatological evaluations, and awareness of atypical presentations are crucial for improving outcomes in individuals with albinism.

## CRedit authorship contribution statement

Musab Ali and mohmmed Elamin generation of the study concept.

Khalid Elsir Ahmed writing of the paper.

Osama and Abdulah review of the paper.

## Consent

informed consent was obtained from the patient for publishing this report.

## Ethical approval

were obtained from ethical community.

## Guarantor

Musab Ali

## Source of funding

None.

## Declaration of competing interest

None existed.
